# Coexistence of turbulence-like and glassy behaviours in a photonic system

**DOI:** 10.1038/s41598-018-35434-z

**Published:** 2018-11-19

**Authors:** Iván R. R. González, Ernesto P. Raposo, Antônio M. S. Macêdo, Leonardo de S. Menezes, Anderson S. L. Gomes

**Affiliations:** 1 Laboratório de Física Teórica e Computacional, Departamento de Física, Universidade Federal de Pernambuco, Recife-PE, 50670-901 Brazil; 2 Departamento de Física, Universidade Federal de Pernambuco, Recife-PE, 50670-901 Brazil

## Abstract

Coexistence of physical phenomena can occur in quite unexpected ways. Here we demonstrate the first evidence in any physical system of the coexistence in the same set of measurements of two of the most challenging phenomena in complex systems: turbulence and spin glasses. We employ a quasi-one-dimensional random fibre laser, which displays all essential ingredients underlying both behaviours, namely disorder, frustration and nonlinearity, as well as turbulent energy cascades and intermittent energy flux between fluctuation scales. Our extensive experimental results are theoretically supported by a newly defined photonic Pearson correlation coefficient that unveils the role of the intermittency and describes remarkably well both the spin-glass Parisi overlap parameter and the distribution of turbulent-like intensity increments. Our findings open the way to unravel subtle connections with other complex phenomena, such as disordered nonlinear wave propagation, Lévy statistics of intensity fluctuations, and rogue waves.

## Introduction

Turbulence is a long-standing challenging phenomenon that occurs in diverse systems, such as fluids^1^ and Bose-Einstein condensates^2^. In the photonic context, turbulent properties have been reported in fibre lasers^3^ and Raman fibre lasers^4,5^, as well as in strongly-disordered random fibre lasers^6,7^. It is important to remark that in ref.^6^ the authors employed a model based on a wave kinetic approach, which is similar to wave turbulence, whereas in^5^ the experimental results are supported by numerical simulations based on the generalized Schrödinger equation. Kolmogorov’s landmark approach to turbulence is based on two principles^1^, namely the energy cascade, whereby energy is transferred from large to small scales until dissipation by viscous forces, and the intermittence of the energy flux between significative scales, which leads to non-Gaussian statistics of the relevant signal, e.g., the velocity increments in a turbulent flow.

On the other hand, the understanding of the spin glass phenomenon still remains elusive, though disorder and frustration have been long recognized as essential underlying ingredients^8^. At low temperatures, spins in a strongly disordered magnetic system ‘freeze’ along random directions. The free energy landscape displays many local minima that can trap the system for a long time. This scenario led Parisi to propose^8^ in 1979 the seminal idea of replica symmetry breaking (RSB), in which identically-prepared spin-glass configurations (i.e., system replicas) occupying different energy minima can give rise to distinct observable measurements.

Remarkably, it was only in 2015 that the first direct experimental evidence of RSB was reported^9^ (see also^10–16^). The experimental system was a random laser in the photonic spin glass phase, in which the coherent oscillation of the modes is frustrated, though keeping nontrivial correlations^17^. A random laser is a cavity-less disordered complex system with a gain medium embedded in a scattering material^18,19^. Laser action occurs due both to the population inversion by suitably pumping the gain medium and the optical feedback arising from the scattering material, as theoretically proposed by Lethokhov^20^ and first experimentally demonstrated by Lawandy and co-workers^21^. The quasi-one-dimensional version of the random lasers is the random fibre laser (RFL)^22^. Random lasers and RFLs thus contrast markedly with conventional lasers, in which the laser oscillation and optical feedback are sustained by fixed mirrors in a closed cavity.

A long-standing open question is whether an intrinsic connection between turbulence and spin glass exists. The first evidence of such connection appeared when approaching turbulence and interface dynamics through the Burgers equation (see, e.g., ref.^23^). In that context, the Kardar-Parisi-Zhang equation, a variant of Burgers equation, bridges the gap between hydrodynamics and disordered systems by providing the system’s partition function in terms of the velocity field, the relevant quantity in fluid turbulence. Addressing this problem for disordered systems was possible under an approach that applied Parisi’s RSB mechanism^23^. More recently, evidence of RSB was reported^24^ in disordered nonlinear wave propagation, which admits^4^ a statistical mechanics description in terms of wave turbulence. These findings suggest the existence of an interplay between turbulence and spin glass that possibly hides a number of striking intertwines at a deeper structure level.

Here we employ a RFL system as the photonic platform to demonstrate the first experimental evidence in any physical system of the coexistence of turbulence-like and spin glass behaviours from the same set of measurements. Our theoretical analysis introduces a novel photonic Pearson correlation coefficient that describes remarkably well both the spin-glass Parisi overlap parameter and the distributions of turbulent-like increments arising from a hierarchical model for the multiscale intensity fluctuations. Moreover, the Pearson coefficient also unveils the role of the intermittence to the RFL properties.

## Results

**Figure 1:**
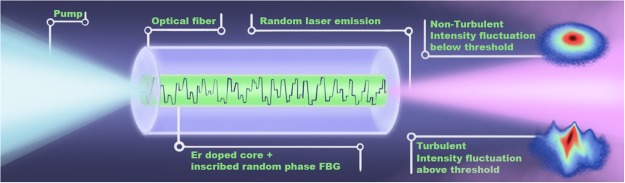
The photonic random fibre laser system. Pictorial description of the 30 cm long erbium-doped RFL with a large number (~10^3^) of specially-designed random fibre Bragg gratings inscribed in the core. The pump source was a semiconductor laser operating in the CW regime at 1480.0 nm. The multimode RFL emission peaks at 1540.0 nm. A rather extensive number of 150,000 spectra were collected at the excitation power above the RFL threshold, *P*/*P*_*th*_ = 2.92. Below the threhsold, a replica-symmetric prelasing phase sets in with non-turbulent behaviour. In contrast, at *P*/*P*_*th*_ = 2.92 the RFL system exhibits the coexistence of photonic RSB spin glass and turbulence-like behaviours in the distribution of intensity increments between successive spectra.

The RFL features and experimental details are described in the Methods section. Figure 1 shows a pictorial illustration of the multimode RFL system pumped by a continuous-wave (CW) semiconductor laser. We remark that an exceptionally large number (~10^3^) of random Bragg gratings were inscribed in the erbium-doped fibre core to strengthen the degree of quenched disorder^25^. The Bragg gratings play the role of quenched random scatterers. This feature makes the RFL to differ substantially from conventional (i.e., not disordered) fibre lasers, in which light performs regular round trips^3^. The nonlinearity arises from the gain provided by the erbium ions, whereas frustration occurs due to the nonlinear interactions of the modes in the strongly disordered fibre medium.

The CW character of the pump source allows for a continuous exploration of the free-energy landscape, particularly in the photonic spin-glass phase above threshold, with the rugged multi-valley structure yielding the correlated states to be sequentially generated through the jump dynamics over energy barriers. Such continuous path can be hampered in the case of pulsed pumping, in which correlations are among states resulting from distinct laser shots. In addition, the laser shots may also hamper the intermittency effect observed in the intensity increments between subsequent spectra that gives rise to a turbulent-like regime in a CW pumped RFL system^7^. Moreover, this erbium-based RFL system provided the first observation^7^ of the statistical signatures of turbulent-like emission in a CW-pumped disordered random laser. Indeed, in ref.^4^ the authors found turbulent emission in quasi-CW Raman fibre lasers in the absence of any form of built-in disorder.

An extensive number (*N*_*s*_ = 150,000) of emission spectra were collected for a value of the excitation power above the random-lasing threshold (*P*/*P*_*th*_ = 2.92). A very long series of output intensities {*I*_*α*_(*k*)}, $\alpha =\mathrm{1,...,}{N}_{s}$, was thus generated for each one of the 512 wavelengths indexed by *k* in the interval [1489.0 nm, 1591.4 nm] around the peak emission at 1540.0 nm. We notice that *α* can be also related to the time variable *t* = *αt*_0_, where *t*_0_ = 100 ms is the integration time between two consecutive spectra acquisitions.

For *P*/*P*_*th*_ = 2.92 the RFL exhibits a photonic spin glass phase with RSB^11,12^ and turbulent-like signature in the distribution of successive intensity increments^7^. The central novelty of the present work lies in the discovery and theoretical characterization, in remarkable agreement with the experimental data, of the coexistence of turbulence-like and spin glass behaviours in the same set of measurements in the RFL system, motivated by the long-standing question in the literature^23^.

Before performing the experimental analysis, we first describe our theoretical framework. The photonic RSB spin glass behaviour is characterized by the distribution *P*(*q*) of values of the Parisi overlap parameter^9^,

1 $${q}_{\alpha \beta }=\frac{\sum _{k}\,{{\rm{\Delta }}}_{\alpha }(k){{\rm{\Delta }}}_{\beta }(k)}{\sqrt{\sum _{k}\,{{\rm{\Delta }}}_{\alpha }^{2}(k)}\sqrt{\sum _{k}\,{{\rm{\Delta }}}_{\beta }^{2}(k)}},$$

where $\alpha ,\beta =\mathrm{1,}\,\mathrm{...,}\,{N}_{s}$ denote the replica labels, the average intensity at the wavelength indexed by *k* is $\bar{I}(k)={\sum }_{\alpha }\,{I}_{\alpha }(k)/{N}_{s}$, and the intensity fluctuation is ${{\rm{\Delta }}}_{\alpha }(k)={I}_{\alpha }(k)-\bar{I}(k)$. Each emission spectrum defines a replica, i.e., a copy of the RFL system under fairly identical experimental conditions. In the photonic RSB spin glass state for *P*/*P*_*th*_ > 1, *P*(*q*) displays a bimodal profile^8,9^ with two maxima around *q* = ±1, whereas in the replica-symmetric prelasing phase for $P/{P}_{th} < 1$ a single maximum occurs at *q* = 0. As replicas with *any* ‘temporal’ separation *τ* ≡ *β* − *α* are considered in (1), thus *P*(*q*) is *not* appropriate to describe photonic turbulence.

On the other hand, if intensity increments are defined for *fixed τ* and *k* as *δI*_*ατ*_(*k*) ≡ *I*_*α*+*τ*_(*k*) − *I*_*α*_(*k*), then the distributions *P*(*δI*_*τ*_) can be used to infer the photonic turbulent-like state at time scale *τ* (here we focus on a wavelength around the peak emission), as explained in the following. In homogeneous and isotropic fluid turbulence, the distribution of velocities is Gaussian, but the distribution of velocity *increments* between different points in the fluid can be Gaussian or not, depending on whether their spatial separation is, respectively, large or short scale^1^. In the latter, the non-Gaussianity of the distribution of velocity increments, evidenced by the presence of heavy tails, provides the signature of the turbulent state. Similarly, in the present photonic RFL system the distribution of intensities is Gaussian^12^ for *P*/*P*_*th*_ = 2.92. Nevertheless, as we shall see below, the time scale *τ* separating the emission spectra is actually determinant to the behaviour of the distribution of intensity *increments* as being Gaussian (*τ* ≫ 1) or not (*τ* ≈ 1). In particular, we show in what follows that this non-Gaussian behaviour of the RFL increments distribution can be actually described remarkably well by a statistical turbulence model that accommodates relevant ingredients of the fluid turbulence phenomenon, such as intermittency and energy fluxes (see below).

In this context, we start by assuming^7^ the existence of *N* relevant time scales of RFL intensity fluctuations, with an intermittent flux of electromagnetic energy between them. The increments distribution *P*(*δI*_1_) between successive spectra at the shortest time scale *τ* = 1 can be calculated exactly from a stochastic model^7,26,27^ that considers a hierarchy of these *N* coupled time scales (see Methods for details), similarly to Kolmogorov’s turbulence mechanisms of energy cascade and intermittency^1^. Noticeably, at small intervals of the long time series {*I*_*α*_(*k*)} we observe that the intensity increments *δI*_*ατ*_ are Gaussian distributed with variance *ε*_*τ*_ in a *local* scale, thus defining a *conditional* Gaussian density *P*(*δI*_*τ*_|*ε*_*τ*_). Since the intermittency effect leads to a slowly fluctuating local variance *ε*_*τ*_ with distribution *f*(*ε*_*τ*_), then *P*(*δI*_1_) can be obtained from the superposition integral

2 $$P(\delta {I}_{1})={\int }_{0}^{\infty }\,P(\delta {I}_{1}|{\varepsilon }_{1})f({\varepsilon }_{1})d{\varepsilon }_{1}\mathrm{.}$$

The solution of the *N*-scale hierarchical model is^7,26,27^ (see Methods section)

3 $$P(\delta {I}_{1})=\frac{{\omega }^{\mathrm{1/2}}}{\sqrt{2\pi {\varepsilon }_{0}}{\rm{\Gamma }}({\boldsymbol{\beta }})}{G}_{\mathrm{0,}N+1}^{N+\mathrm{1,0}}(\begin{array}{c}-\\ {\boldsymbol{\beta }}-\mathrm{1/2,0}\end{array}|\frac{\omega {(\delta {I}_{1})}^{2}}{2{\varepsilon }_{0}}),$$

where *G* denotes the Meijer-*G* function^28^, *ε*_0_ is the variance at the largest scale, *β* essentially represents the ratio between the deterministic and stochastic model coefficients, ${\boldsymbol{\beta }}\equiv (\beta ,\mathrm{...},\beta )$ is an *N*-component vector, *ω* = *β*^*N*^, and Γ(***β***) = [Γ(*β*)]^*N*^ is a product of *N* Γ-functions. We observe from equation (3) that the ratio (2*ε*_0_/*ω*)^1/2^ sets a typical scale for the distribution of intensity fluctuations *δI*_*τ*_ with *τ* = 1. As we shall see below, one feature of the turbulence-like behaviour is the large-*δI*_1_ asymptotic behaviour of *P*(*δI*_1_), which displays non-Gaussian decay for *τ* = 1. In contrast, for *τ* ≫ 1 a Gaussian *P*(*δI*_*τ*_) emerges, consistently with the non-turbulent state observed at large time scales.

Although this approach is appropriate to describe photonic turbulence, it is, however, unsuitable to signalize the spin glass phenomenon. Therefore, in order to capture the interplay of both turbulence-like and spin glass behaviours, we first need to define the new variable *y*_*ατ*_(*k*) ≡ *I*_*α*+*τ*_(*k*) − *cI*_*α*_(*k*), where *c* = 0 if *τ* = 0 and *c* = 1 if *τ* ≥ 1. Its fluctuation around the average is ${\delta }_{\alpha \tau }(k)={y}_{\alpha \tau }(k)-{\bar{y}}_{\tau }(k)$. For *τ* = 0 this fluctuation coincides with that of the Parisi overlap parameter (1), i.e., *δ*_*ατ*__=0_(*k*) = Δ_*α*_(*k*). We thus introduce the photonic Pearson coefficient to measure the correlation between *δ*-fluctuations at a fixed *τ*:

4 $${Q}_{\alpha \beta ,\tau }=\frac{\sum _{k}\,{\delta }_{\alpha \tau }(k){\delta }_{\beta \tau }(k)}{\sqrt{\sum _{k}\,{\delta }_{\alpha \tau }^{2}(k)}\sqrt{\sum _{k}\,{\delta }_{\beta \tau }^{2}(k)}}\mathrm{.}$$

The introduction of this new Pearson correlation coefficient is actually a key contribution of our work, since it is simultaneously sensitive to both turbulence-like and spin glass behaviours. Indeed, for *τ* = 0 it recovers the Parisi overlap parameter of the photonic glassy phase, *Q*_*αβ*,*τ*__=0_ = *q*_*αβ*_. On the other hand, for *τ* ≥ 1 the Pearson coefficient is able to infer the turbulence phenomenon. For example, the maximum turbulence-like signature (intermittency) is manifested at the shortest time scale *τ* = 1. In contrast, for *τ* ≫ 1 the short-time-scale effects and intermittency rapidly fade away and a crossover takes place to the non-turbulent (Gaussian) behaviour, as demonstrated below.

We now turn to the analysis of the experimental RFL data. In the following, we define the normalized intensity increments as ${x}_{\alpha \tau }(k)=\delta {I}_{\alpha \tau }(k)/[{\sum }_{\alpha }\,\delta {I}_{\alpha \tau }^{2}(k{)]}^{\mathrm{1/2}}$.

**Figure 2:**
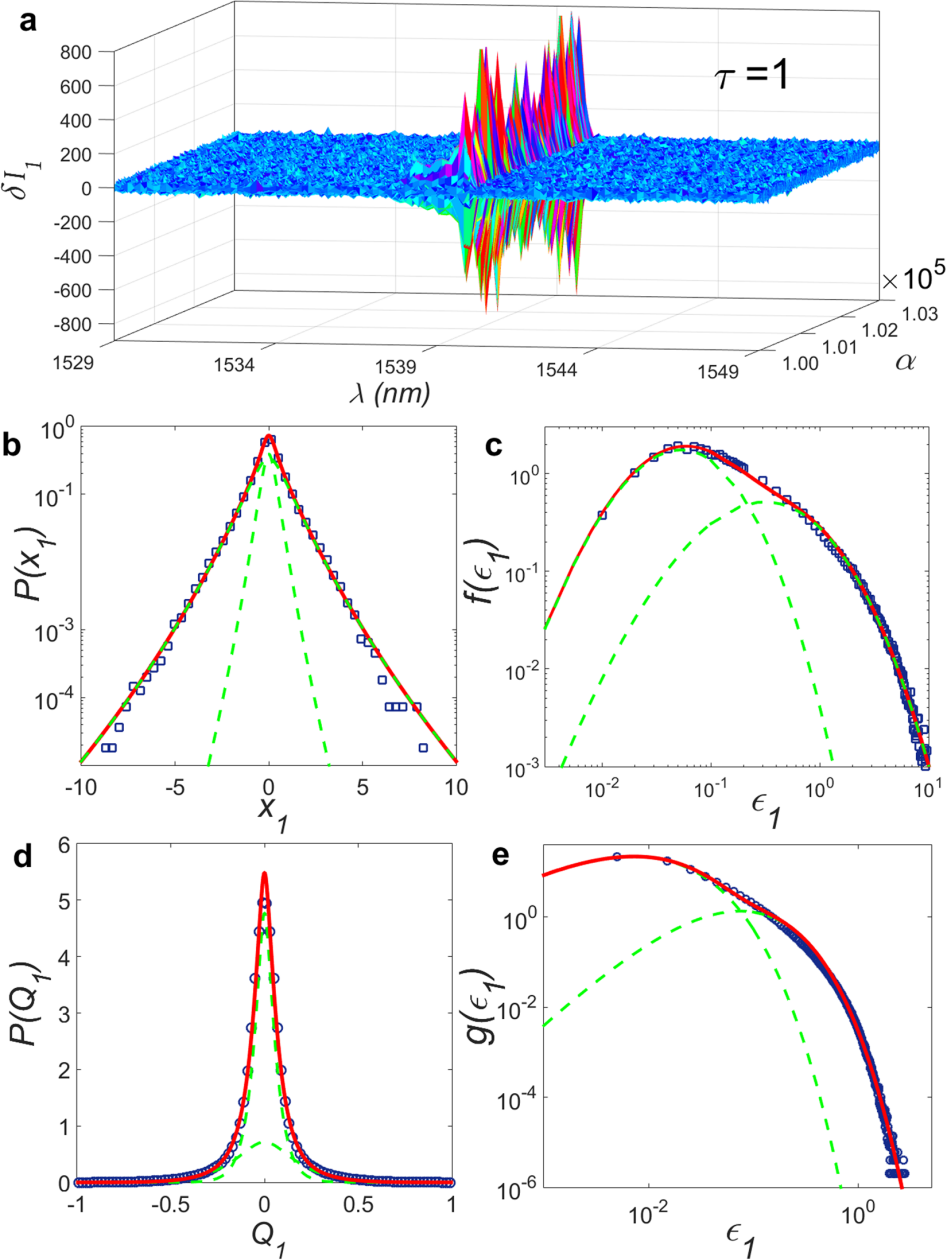
Photonic turbulence and intermittency effect for short separation time scale between RFL spectra (*τ* = 1). (**a**) Plot of 3000 experimental intensity increments, *δI*_*ατ*_(*k*) = *I*_*α* + *τ*_(*k*) − *I*_*α*_(*k*), between successive spectra (*τ* = 1) as a function of the spectrum label *α* and wavelength *λ* indexed by *k* in the interval [1529.0 nm, 1549.0 nm]. (**b**) Distribution *P*(*x*_*τ*_), *τ* = 1, of normalized increments ${x}_{\alpha \tau }(k)=\delta {I}_{\alpha \tau }(k)/[{\sum }_{\alpha }\,\delta {I}_{\alpha \tau }^{2}(k{)]}^{\mathrm{1/2}}$, at the wavelength *λ* = 1539.8 nm around the peak emission, obtained from the whole set of 150,000 spectra (blue squares). The large deviation from the parabolic shape observed in this semi-log plot is consistent with the non-Gaussian photonic turbulent-like behaviour of *P*(*x*_1_), which arises for this maximum intermittency state at *τ* = 1. The red solid line displays the nice fit to the results of the hierarchical model, with *P*(*x*_1_) given by a statistical mixture of two Meijer-*G* functions with *N* = 6 intensity fluctuation time scales. (**c**) Associated variance distribution *f*(*ε*_1_) from which *P*(*x*_1_) is obtained via the superposition integral (2). Experimental data (blue squares) are also nicely fitted by the theoretical result (red solid line). The individual mixture components are shown by green dashed lines in (**b**) and (**c**). (**d**) Distribution *P*(*Q*_1_) of values of the photonic Pearson coefficient and (**e**) associated variance distribution *g*(*ε*_1_) calculated from 10,001 spectra. The model results for *N*_*Q*_ = 2 (red solid lines) also adjust remarkably well to the experimental data (blue circles), with the mixture components shown in green dashed lines.

For the shortest time scale *τ* = 1, Fig. 2(a) shows an excerpt of the large dataset, with 3000 (out of 150,000) intensity increments between successive spectra. The distribution *P*(*x*_1_) at *λ* = 1539.8 nm around the peak emission, calculated from the whole dataset, is depicted in squares in Fig. 2(b). In this semi-log plot, the large deviation from the parabolic shape signalizes the non-Gaussian turbulence-like behaviour of *P*(*x*_1_). The red solid line shows a remarkable agreement with the results of the hierarchical model. The best fit is obtained for *N* = 6 time scales of intensity fluctuations, with *P*(*x*_1_) given by a statistical mixture of two functions, *P*_*a*_(*x*_1_) and *P*_*b*_(*x*_1_), given by equation (3) for *x*_1_: *P*(*x*_1_) = *pP*_*a*_(*x*_1_) + (1 − *p*) *P*_*b*_(*x*_1_), with parameters *p* = 0.30, *β*_*a*_ = 8.53, *β*_*b*_ = 6.47, *ε*_0,*a*_ = 0.16 and *ε*_0,*b*_ = 1.36. Moreover, each mixture component displays non-Gaussian asymptotic behaviour in the form of a heavy stretched-exponential tail, consistently with the turbulent-like state (see Methods).

Figure 2(c) presents the associated variance distribution *f*(*ε*_1_) which, in accordance with equation (2), is also given by a statistical mixture with the same parameters as *P*(*x*_1_). Remarkably, as seen in Fig. 2(c) the effect of the small and large variances *ε*_1_ are distinguishably incorporated by each mixture component. Statistical mixtures can be also found, e.g., in oceanic turbulence, due to the combination of active and quiescent modes in the distribution of kinetic energy dissipation rates^29^. In the present work we do not attempt to separate our experimental results according to some identified mechanism responsible for the statistical mixtures, but we infer from general grounds that such mechanism, in the presence of both nonlinearity and disorder, could arise from a subtle combination of stimulated and spontaneous turbulent emissions.

On the other hand, by considering a hierarchical model similar to that described in the Methods section for the intensity increments, the distribution *P*(*Q*_1_) of the Pearson coefficient for *τ* = 1 can be also calculated via a superposition integral as equation (2), yielding

5 $$P({Q}_{\tau })=\frac{c}{\mathrm{(1}-{Q}_{\tau }^{2})}{G}_{\mathrm{0,}{N}^{(Q)}+1}^{{N}^{(Q)}+\mathrm{1,0}}(\begin{array}{c}-\\ {{\beta }}^{(Q)}-\mathrm{1/2,}\,0\end{array}|\frac{{\omega }^{(Q)}{z}_{\tau }^{2}}{2{\varepsilon }_{0}^{(Q)}}),$$

with *z*_*τ*_ = 2arctanh(*Q*_*τ*_), ${c}^{2}=4{\omega }^{(Q)}\mathrm{/[2}\pi {\varepsilon }_{0}^{(Q)}{{\rm{\Gamma }}}^{2}({{\beta }}^{(Q)})]$, and ${\varepsilon }_{0}^{(Q)}$, *ω*^(*Q*)^ and *β*^(*Q*)^ defined similarly to equation (3).

In Fig. 2(d,e) the experimental data (circles) are plotted together with the best theoretical fits (red lines) for *P*(*Q*_1_) and the associated variance distribution *g*(*ε*_1_). A very nice agreement is observed for *N*^(*Q*)^ = 2 time scales of the Pearson coefficient and parameters of a similar statistical mixture: *p*^(*Q*)^ = 0.70, ${\beta }_{a}^{(Q)}=2.53$, ${\beta }_{b}^{(Q)}=3.50$, ${\varepsilon }_{\mathrm{0,}a}^{(Q)}=0.03$ and ${\varepsilon }_{\mathrm{0,}b}^{(Q)}=0.18$. In particular, we argue below that the unimodal behaviour of *P*(*Q*_1_) is intrinsically related to the maximum intermittency effect underlying the turbulent-like state for *τ* = 1.

**Figure 3:**
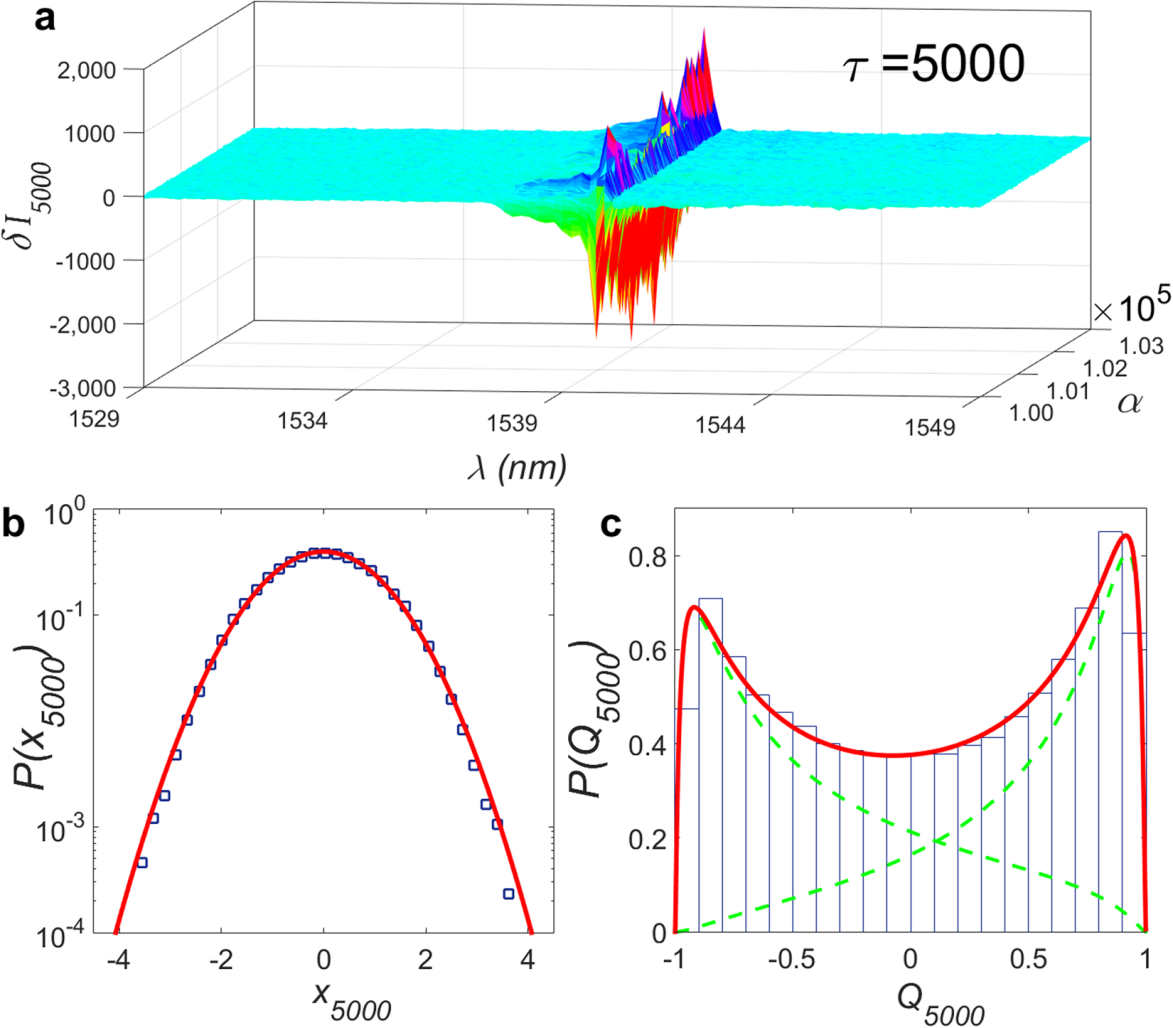
Photonic spin glass behaviour and suppression of intermittency for large separation time scale between RFL spectra (***τ*** = 5000). (**a**) Plot of 3000 experimental intensity increments, *δI*_*ατ*_(*k*) = *I*_*α* + *τ*_(*k*) − *I*_*α*_(*k*), between spectra separated by *τ* = 5000 time units, as a function of the spectrum label *α* and wavelength *λ* indexed by *k* in the interval [1529.0 nm, 1549.0 nm]. (**b**) Semi-log plot of the distribution *P*(*x*_*τ*_), *τ* = 5000, of normalized increments, at the wavelength *λ* = 1539.8 nm around the peak emission, obtained from the whole set of 150,000 spectra (blue squares). The red solid line displays the nice fit to the results of the hierarchical model. At the large time scale *τ* = 5000, the parabolic shape indicates that the distribution *P*(*x*_5000_) assumes a Gaussian form (*N* = 0 in the theoretical model), showing that intermittency has been fully suppressed. (**c**) Histogram with the distribution *P*(*Q*_5000_) of values of the photonic Pearson coefficient calculated from 10,001 spectra displaying a spin-glass-like bimodal profile. The experimental histogram is also nicely fitted by the theoretical model (red solid line), with the mixture components of *P*(*Q*_5000_) depicted by green dashed lines. Since the intermittency rapidly fades away for *τ* > 1, then *P*(*Q*_5000_) appears qualitatively similar to the distribution *P*(*q*) of the Parisi overlap parameter (1) that characterizes the spin glass behaviour.

A remarkably distinct picture emerges for large separation time scales between the emission spectra, *τ* ≫ 1. Indeed, Fig. 3 displays the crossover to the non-turbulent behaviour for *τ* = 5000. The intensity increments at the wavelength *λ* = 1539.8 nm now assume a Gaussian distribution *P*(*x*_5000_), Fig. 3(b), indicating that intermittency has been fully suppressed (i.e., *N* = 0 in the theoretical model). Concurrently, a bimodal profile of *P*(*Q*_5000_) is observed in Fig. 3(c), showing a significant contrast with the unimodal profile of *P*(*Q*_1_) that can be understood as follows. The intermittency effect tends to increase for short scales the probability of events that are rarer at much larger scales. As seen in Fig. 3(c), when intermittency is absent in the non-turbulent state for *τ* = 5000, values *Q*_5000_ ≈ 0 are less probable than those around *Q*_5000_ = ± 1. Consequently, when intermittency is maximum for *τ* = 1, values *Q*_1_ ≈ 0 have their probability much increased, leading to the unimodal behaviour of *P*(*Q*_1_) in the turbulent-like state. (The unimodal behaviour of *P*(*Q*_1_) in the RSB spin glass phase of the RFL at *P*/*P*_*th*_ = 2.92 should not be confused with the unimodality of *P*(*q*), which, instead, signals the prelasing phase with replica symmetry for *P*/*P*_th_ < 1).

Finally, we also observe that the bimodal behaviour of *P*(*Q*_5000_) resembles the spin-glass profile^11^ of the distribution *P*(*q*) of the Parisi overlap parameter (1) for *P*/*P*_*th*_ > 1. In fact, since the Parisi overlap parameter considers *all* separation times *τ* between spectra, we thus expect that the statistical weight of the replica overlaps with *τ* ≫ 1 dominates over the long time series. As a consequence, *P*(*Q*_*τ*_) for *τ* ≫ 1 should actually appear qualitatively similar to the distribution *P*(*q*) that characterizes the RSB spin glass state.

We lastly remark that Figs 2 and 3 are actually insensitive to the number of RFL modes collected, since the distributions were calculated for the single mode at *λ* = 1539.8 nm. Moreover, we have also confirmed the stability of the photonic spin-glass results regarding the number of modes around the peak emission in Eq. (1).

## Discussion

The coexistence phenomenon addressed in this work relates two of the most challenging behaviours of complex systems: turbulence and spin glass. We highlight that the remarkable agreement between the RFL experimental data and the theoretical results was possible only due to the very large dataset analysed (>10^5^ RFL emission spectra), combined with the suitable definition of a new photonic Pearson coefficient to simultaneously account for both phenomena.

We also remark that since both photonic regimes of spin glass and turbulence have been separately identified^7,11,12^ in all excitation powers studied above the RFL threshold, therefore we believe that the coexistence phenomenon probed in the present work at *P*/*P*_*th*_ = 2.92 is also robust for other values of *P*/*P*_*th*_ above threshold.

We hope that our findings open the way to investigations on possible unforeseen coexistence of other related complex phenomena that share similar mechanisms, including Lévy statistics of intensities^16,30^, disordered nonlinear wave propagation^24,31^, and rogue waves^32,33^.

## Methods

### Experimental characterization

The RFL system is a 30 cm long polarization maintaining erbium-doped structure^25^ fabricated by CorActive (peak absorption of 28 dB/m at 1530.0 nm, mode field diameter of 5.7 *μ*m, 0.25 numerical aperture), see Fig. 1. By applying a specially-designed interferometric technique, a fibre Bragg grating with random phase was written over the whole fibre. Each fibre grating acts as a quenched scatterer and an exceptionally high number of scatterers (~10^3^) was inscribed in the fibre to improve the disorder strength. The pump source was a home-assembled CW semiconductor laser operating at 1480.0 nm and presenting two longitudinal modes, as determined from speckle measurements. The linewidths were measured to be 0.70 nm using an optical spectrum analyzer (spectral resolution of 0.06 nm). Random laser emission occurs for input powers above the threshold value, *P*_*th*_ = (16.30 ± 0.05) mW. We have also ensured^12^ that the pump laser fluctuations do not affect the erbium-based RFL fluctuations. An instrument-limited laser linewidth of 0.2 nm was determined above threshold. Although this is a relatively narrow linewidth, the RFL was shown^11^ to emit a large number of longitudinal modes (~200) from measurements using the speckle contrast technique. Although our RFL operates in a single transverse mode regime, the presence of a several longitudinal modes in the 30 cm length RFL confers its quasi-one-dimensional character^25^.

A rather extensive number (*N*_*s*_ = 150,000) of emission spectra were collected for the excitation power *P*/*P*_*th*_ = 2.92. A spectrometer with a nominal 0.2 nm resolution at 1530.0 nm was employed, with a liquid-N_2_-cooled infrared CCD camera as the detector. A very long series of output intensities {*I*_*α*_(*k*)}, $\alpha =\mathrm{1,}\,\mathrm{...,}\,{N}_{s}$, was generated for each one of the 512 wavelengths indexed by *k* in the interval *λ* ∈ [1489.0 *nm*,1591.4 *nm*] around the peak emission at 1540.0 nm. The integration time between two consecutive spectra acquisitions is *t*_0_ = 100 ms. As the lifetime of the active erbium ions in the RFL is of order of hundreds of *μ* s or less, then this integration time actually corresponds to averaging over many lasing emissions. We comment that this averaging process does not invalidate our method since it is the definition and characterization of the experimental replicas resulting from these many lasing emissions that actually matter to describe the emergence of distinct photonic regimes (see, also^9,14,15^, where similar averaging phenomena occur).

### Hierarchical model

Here we review on the stochastic hierarchical model that has been successfully applied to describe turbulence-like properties in diverse systems, such as fluids^26,27^, financial markets^26,27^, and a photonic RFL^7^.

In fluid turbulence, the significative statistical quantities are the velocity increments between two close points in the flow^1^. By analogy, in photonic turbulence we may consider intensity increments between successive optical spectra, *δI*_*ατ*_(*k*) ≡ *I*_*α*+*τ*_(*k*) − *I*_*α*_(*k*), with *τ* = 1 (shortest separation time scale between the spectra $\alpha =\mathrm{1,}\,\mathrm{...,}\,{N}_{s}$) and *k* denoting the wavelength index. In the prelasing regime (*P*/*P*_*th*_ < 1), in which nonlinearities are irrelevant, the intensity increments are statistically independent and the probability distribution for a given wavelength, *P*(*δI*_*τ*_), *τ* = 1, is a Gaussian. In contrast, when the excitation power is increased beyond the threshold, nonlinearities give rise to a turbulent-like emission in which the Gaussian form of the intensity increments distribution remains valid *only at a local level*, with a slowly fluctuating variance *ε*_*τ*_ for *τ* = 1. Therefore, the local *conditional* distribution at *τ* = 1 can be written for *P*/*P*_*th*_ > 1 as $P(\delta {I}_{1}|{\varepsilon }_{1})=\exp [\,-\,{(\delta {I}_{1})}^{2}\mathrm{/2}{\varepsilon }_{1}]/\sqrt{2\pi {\varepsilon }_{1}}$. Consequently, in the statistical description of turbulence the non-Gaussian global form of *P*(*δI*_1_) can be obtained by compounding the local Gaussian *P*(*δI*_1_|*ε*_1_) with a background distribution of variance fluctuations *f*(*ε*_1_), in the form of the superposition integral (2).

The background density *f*(*ε*_1_) can be obtained exactly from a hierarchical stochastic model^7,26,27^ that accommodates the basic concepts of Kolmogorov’s turbulence theory^1^, namely energy cascade (the transfer of energy from large to small scales) and intermittency, which is caused by fluctuations in the energy transfer rates between relevant adjacent scales. Intermittency can be understood as the tendency of the distributions of the relevant variable, say the fluid velocity or intensity increments, to change substantially as one moves from a large scale, where inertial effects dominate, to a small scale, in which dissipation becomes dominant. Our hierarchical model is defined^7,26,27^ by the following set of stochastic differential equations,

6 $$d{\varepsilon }^{(i)}(t)=-{\gamma }_{i}({\varepsilon }^{(i)}-{\varepsilon }^{(i-\mathrm{1)}})dt+{\kappa }_{i}\sqrt{{\varepsilon }^{(i)}{\varepsilon }^{(i-\mathrm{1)}}}d{W}^{(i)}(t),$$

for $i=\mathrm{1,}\,\mathrm{...,}\,N$, where in the photonic context *N* is the number of relevant time scales of intensity fluctuations. The variable *ε*^(*i*)^ represents the fluctuating variance parameter at the respective scale in the hierarchy. In our notation, the *N*-th hierarchy level is assigned to the shortest time scale *τ* = 1, that is, *ε*^(*i* = *N*)^↔*ε*_*τ*__=1_, whereas *ε*_0_≡*ε*^(0)^ is related to the largest time scale. The coefficients *γ*_*i*_ and *κ*_*i*_ are positive and *dW*^(*i*)^ denote independent Wiener processes. The first term in (6) describes the deterministic coupling between adjacent scales, whilst the stochastic term accounts for the non-linear couplings with the background variables at all scales, setting the ultimate source of intermittency. For convenience, we define the dimensionless parameter $\beta =2{\gamma }_{i}/{\kappa }_{i}^{2}$, so that (2*ε*_0_/*β*^*N*^)^1/2^ determines a typical scale for the distribution *P*(*δI*_*τ*_) of intensity fluctuations for *τ* = 1; see equation (3).

Under the assumption of large time scales separation, ${\gamma }_{N}\gg {\gamma }_{N-1}\gg \ldots \gg {\gamma }_{1}$, we write the density *f*(*ε*_1_) ≡ *f*(*ε*^(*N*)^) as

7 $$f({\varepsilon }^{(N)})=\int \,d{\varepsilon }^{(N-\mathrm{1)}}\mathrm{...}\int \,d{\varepsilon }^{\mathrm{(1)}}f({\varepsilon }^{(N)}|{\varepsilon }^{(N-\mathrm{1)}}\mathrm{)...}f({\varepsilon }^{\mathrm{(1)}}|{\varepsilon }^{\mathrm{(0)}}),$$

where the conditional distribution *f*(*ε*^(*i*)^|*ε*^(*i*−1)^) arises from the stationary solution of (6) in the form of a gamma distribution,

8 $$f({\varepsilon }^{(i)}|{\varepsilon }^{(i-\mathrm{1)}})=\frac{{(\beta /{\varepsilon }^{(i-\mathrm{1)}})}^{\beta }}{{\rm{\Gamma }}(\beta )}{({\varepsilon }^{(i)})}^{\beta -1}{e}^{-\beta {\varepsilon }^{(i)}/{\varepsilon }^{(i-\mathrm{1)}}}.$$

Remarkably, the multiple integral in equation (7) has an exact analytical representation in terms of a Meijer *G*-function^28^,

9 $$f({\varepsilon }_{1})=\frac{\omega }{{\varepsilon }_{0}{\rm{\Gamma }}({\boldsymbol{\beta }})}{G}_{\mathrm{0,}N}^{N\mathrm{,0}}(\begin{array}{c}-\\ {\boldsymbol{\beta }}-1\end{array}|\frac{\omega {\varepsilon }_{1}}{{\varepsilon }_{0}}),$$

where $\beta \equiv (\beta ,\,\mathrm{...,}\,\beta )$ is an *N*-component vector, *ω* = *β*^*N*^, and Γ(***β***) = [Γ(*β*)]^*N*^ is a product of *N* Γ-functions. Finally, by substituting *f*(*ε*_1_) and the local conditional Gaussian *P*(*δI*_1_|*ε*_1_) into the superposition integral (2), we obtain equation (3).

We further note that the large-*δI*_1_ asymptotic limit of equation (3) is the heavy-tailed modified stretched exponential^28^,

10 $$P(\delta {I}_{1}) \sim {(\delta {I}_{1})}^{2\theta }\exp [-(N+\mathrm{1)[}\omega {(\delta {I}_{1})}^{2}\mathrm{/2}{\varepsilon }_{0}{]}^{\mathrm{1/(}N+\mathrm{1)}}],$$

where *θ* = *N*(*β* − 1)/(*N* + 1), which displays important deviations from the Gaussian.

## Data Availability

All relevant data are available from the authors.
